# Influence of light, temperature and salinity on dissolved organic carbon exudation rates in *Zostera marina* L.

**DOI:** 10.1186/2046-9063-8-19

**Published:** 2012-08-31

**Authors:** James Kaldy

**Affiliations:** 1 Western Ecology Division, US Environmental Protection Agency, 2111 SE Marine Science Dr, Newport, OR, 97365, USA

**Keywords:** Carbon balance, Seagrass, Exudation, Rhizodeposition, Gradients

## Abstract

**Background:**

Marine angiosperms, seagrasses, are sentinel species of marine ecosystem health and function. Seagrass carbon budgets provide insight on the minimum requirements needed to maintain this valuable resource. Carbon budgets are a balance between C fixation, growth, storage and loss rates, most of which are well characterized. However, relatively few measurements of dissolved organic carbon (DOC) leaf exudation or rhizodeposition rates exist for most seagrass species. Here I evaluate how eelgrass (*Zostera marina* L.) DOC exudation is affected by a single factor manipulation (light, temperature or salinity). Eelgrass plants were hydroponically exposed to treatments in experimental chambers (separate leaf and rhizome/root compartments) with artificial seawater medium. Regression analysis of changes in the DOC concentration through time was used to calculate DOC exudation rates.

**Results:**

Exudation rates were similar across all treatments in all experiments. For all experiments, pooled leaf DOC exudation ranged between 0.032 and 0.069 mg C gdw^-1^ h^-1^, while rhizodeposition ranged between 0.024 and 0.045 mg C gdw^-1^ h^-1^. These rates are consistent with previously published values and provide first-order estimates for mechanistic models.

**Conclusions:**

*Zostera marina* carbon losses from either leaf exudation or rhizodeposition account for a small proportion of gross primary production (1.2-4.6%) and appear to be insensitive to short-term (*e.g.*, hours to days) environmental variations in chamber experiments. Based on these preliminary experiments, I suggest that *Z. marina* DOC exudation may be a passive process and not an active transport process.

## Background

Seagrasses are marine angiosperms that provide valuable ecosystem services and are often described as foundation species or ecosystem engineers [1,2]. Additionally, they have been identified as sentinel species that can indicate marine ecosystem health and function [3]. Therefore, there is interest in using seagrass models to evaluate physiological and ecological effects of stressors such as nutrient loading, light reductions and geochemical toxicity (*e.g.*, sulfides, nitrogen toxicity). Quantitative models focused on the plants’ carbon budgets provide detailed insight into their potential survival as it relates to varying levels of environmental stress [4].

Seagrass carbon budgets like those of all plants are a balance between C fixation, growth, storage and loss rates, in the simplest form:

(1) $$WP=C_{\mathrm{fixed}}-Lf_{\mathrm{resp}}-Lf_{\mathrm{exud}}-RR_{\mathrm{resp}}-RR_{\mathrm{exud}}-Structural$$

Where WP = whole plant, C_fixed_ = photosynthesis, Lf and RR represent leaf and rhizome/root tissues, while subscripts *resp* and *exud* represent respiration and exudation loss terms respectively. Structural materials represent carbohydrates incorporated into cell walls during growth and development. A positive WP indicates surplus carbon that can be stored, while a negative WP indicates a carbon deficit that may be supplied from stored reserves [5,6]. Many studies have focused on the fixation portion of carbon budgets especially with development of commercially available equipment and concomitant cost reductions to measure photosynthetic physiology using oxygen evolution [6-8] and pulse amplitude modulated (PAM) fluorometry [9-11]. Likewise, understanding carbon storage dynamics (*e.g.*, non-structural carbohydrate carbon) provides insight into seagrasses stress tolerance, especially low light stress [12]. Pioneering work conducted during the late 1970’s and early 1980’s suggested that carbon loss via exudation (DOC, dissolved organic carbon) from leaves was small [13-15]. Recent work using compound specific stable isotope analyses could not detect coupling between *Z. marina* production and sediment bacteria [16] suggesting limited carbon exudation. As a result most seagrass studies and models neglect leaf DOC exudation. However, other recent work in tropical and subtropical seagrass systems suggests that DOC exudation can be substantial [17-19]. The contrasting conclusions from these studies and lack of work taking into account variability in environmental conditions suggest that further attention is required to better understand and model these processes.

Seagrass rhizodeposition, release of carbon exudates through rhizomes and roots, is thought to be a relatively minor loss [14,20] and it has been generally ignored in seagrass production models. However, in some seagrass species, rhizodeposition is greater than leaf exudation and can account for 15-30% of primary production [18,21]. Rhizodeposition is used synonymously with Rhizome + Root exudation throughout this document. In terrestrial plants, rhizodeposition can account for up to 17% of primary production and has been shown to fuel soil microbial processes [22]. Likewise, a recent seagrass modeling study found that DOC rhizodeposition rates were a critical parameter for modeling microbially mediated sediment oxygen demand in a subtropical system [23]. Although several studies have estimated DOC exudation and rhizodeposition they have been conducted under static environmental conditions. Variations in exudation rates under fluctuating environmental conditions or across a gradient of conditions may be important constraints for dynamic seagrass production models.

A number of studies have concluded that seagrass derived DOC contributes to the labile autochthonous carbon pool available to heterotrophic bacteria [24-26]. Quantifying seagrass DOC production has generally been carried out using chambers to measure DOC fluxes from intact communities [13,14,17-19,25]. These studies are inherently confounded by DOC exudation from multiple primary producer sources, including microalgal epiphytes, sediment microbial community (which may be heterotrophic or autotrophic) and water column planktonic and microbial communities as well as seagrass production. Using a variety of methods and assumptions, seagrass contribution to DOC efflux can sometimes be partitioned out. However, few if any studies have directly measured seagrass DOC production rates *in vitro* by minimizing the influence of confounding primary producers (*e.g.*, hydroponic chamber experiments), which will have their own unique limitations and caveats. Additionally, there have been no studies that evaluate how DOC loss rates respond to drivers that influence seagrass production.

Light, temperature and salinity are environmental drivers which have potentially large effects on carbon budgets by influencing rate processes and ultimately carbon balance. I predict that seagrass DOC exudation rates will be a function of these environmental drivers. My objectives were to develop a hydroponic chamber system for minimizing the number of DOC sources and to quantify how seagrass DOC exudation and rhizodeposition varied in response to a range of values for single environmental drivers (light, temperature or salinity).

## Methods

### Environmental background

For all experiments, *Zostera marina* plants were collected from Yaquina Bay adjacent to the Hatfield Marine Science Center (HMSC) pump-house dock in Newport, Oregon, USA. The central Oregon coast experiences an “oceanic” or “maritime” climate moderated by the Pacific Ocean, resulting in relatively stable annual temperatures and strong seasonal precipitation patterns [27]. Annually integrated underwater irradiance in the seagrass bed near this site has been measured around 1200 mol photons m^-2^ y^-1^ (~ 3 mol m^-2^ d^-1^) with mean monthly water temperatures ranging between 9 and 13°C [28]. Water column salinity at this site generally ranges between 24 and 34 [18], although wider variations are also common [29]. Eelgrass here is intertidal, extending from *ca.* +0.25 m to −2 m Mean Lower Low Water (MLLW) tidal elevation; plants for these experiments were collected from the subtidal at about -1 m MLLW elevation. Care was taken to excavate the rhizome/root complex with minimal damage. Senescent leaf material was removed and rhizomes were trimmed to five internodes with a razor blade. Previous work [30] has shown that translocation and metabolism decreases with increasing number of rhizome nodes (*e.g.*, increased tissue age), with the first 4 nodes being most physiologically active. Epiphytes were removed by wiping each blade with a wet cloth. This removed most of the epiphyte community with no visible damage to the epidermis and minimal leaf breakage. Plants were held overnight in the dark in flowing seawater prior to initiating the experiments.

### Chambers and measurements

**Figure 1:**
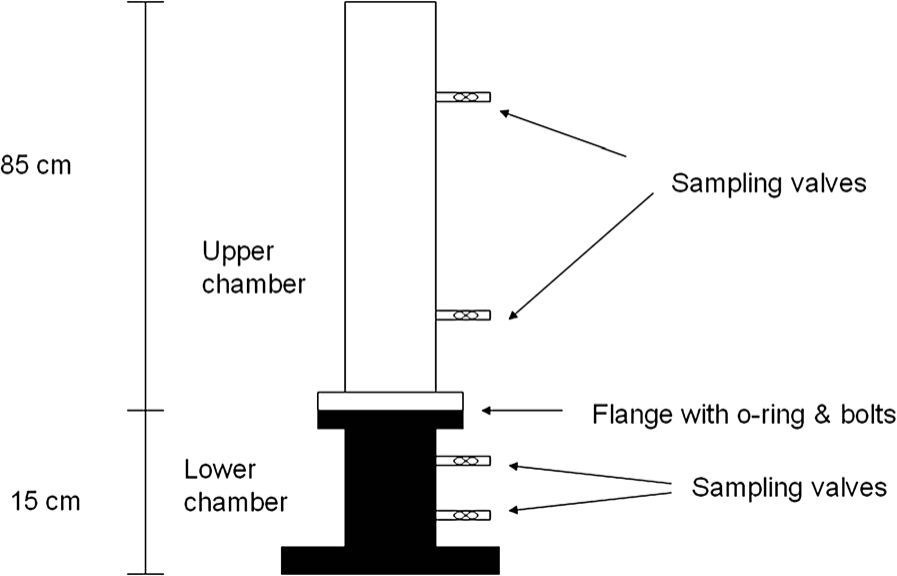
**Schematic drawing of the experimental chambers used in the experiments to hydroponically culture eelgrass for DOC exudation measurements.** Drawing is not to scale.

Fifteen dual-compartment experimental chambers were used to hydroponically incubate *Z. marina* plants under various experimental treatments (Figure 1). Experimental treatments were selected to span the range of field conditions. The upper compartment has a volume of about 6 l and is clear acrylic with two sampling ports. The lower compartment has a volume of 0.8 l and is opaque acrylic also fitted with two sampling ports. Compartments were separated by a bulkhead with a hole and a soft silicone stopper that was slit to accept the plant. The portion of the plant held within the stopper was wrapped with Parafilm® to create a snug fit and the cut was filled with a small piece of “plumbers putty” (Ace Hardware Inc., Oak Brook, Illinois, USA) to maintain a water tight seal. Each chamber was then randomly assigned to a treatment cabinet used to incubate the chambers; each cabinet contained a light source and a polycarbonate tank (60 cm × 60 cm × 90 cm) that was used as a water bath to control temperature. In each experiment, four replicate plants and one control (artificial seawater (AFSW) medium with solid stopper and no plant) were exposed to any given treatment level. Because each cabinet contains all treatment chambers, this design is inherently pseudoreplicated [31]. This could not be avoided given time, financial and logistical constraints (number of chambers and cabinets [32]). Pseudoreplicated statistical designs can provide important information and be used to develop testable hypotheses [32]. Comparison of hydroponic and sediment cultured *Z. marina* have found no statistically significant differences [33], suggesting that hydroponically grown plants respond similarly to those grown in sediments. Light and temperature conditions for each experiment are detailed below. Air was bubbled through each chamber to maintain mixing in the upper compartment and to prevent inorganic carbon limitation. Dye experiments indicated that chamber seals were effective and that bubbling air provided a uniform, well mixed system with no “dead volumes” in ≤10 s [34-36]. 

For each experiment, five 19 liter batches of artificial seawater medium (AFSW) were mixed in pre-combusted glass carboys using Red Sea Salts ® (Red Sea Phish Farms Inc., Eliat, Israel) and Milli-Q water. Each carboy was amended with 200 ml of 2 mM NH_4_Cl to provide a initial NH_4_ concentration of about 20 μM. Previous work indicates that *Z. marina* growing in Yaquina Bay are nutrient replete [18]. Prior to initiating an experiment, triplicate samples (T_0_) were obtained from each carboy to characterize initial DOC and nutrient concentrations. For the purposes of these experiments “low DOC” reflects concentrations < 0.5 mg C l^-1^. DOC samples were collected from both above- and below-ground chamber compartments at 24, 48 and 72 h.

Surface sterilization techniques (*e.g.*, dilute hypochlorite, dilute H_2_O_2_, low pH) were investigated to reduce plant bacterial load; however, these treatments adversely affected the plants causing leaf tissue to turn brown. Other studies have added antibiotic compounds such as erythromycin, penicillin, streptomycin [37,38]; however, this also increases the background DOC concentration. Consequently, no additives were used to control bacteria in these experiments. Mechanical removal of the epiphyte community and an artificial medium were used to minimize the abundance of bacteria. However, uncontrolled bacterial populations likely resulted in erratic DOC concentrations at the later sample times of 48 and 72 h (see Results and Discussion). Consequently, I calculated DOC exudation (described below) over the first 24 h based on the assumption that bacterial populations were minimal during this period since previous work indicates bacteria populations require about one day to double in size [39].

DOC concentrations were measured using high temperature combustion methods with a Shimadzu Total Organic Carbon Analyzer Vcsh (Shimadzu Corp., Kyoto, Japan). Water samples were obtained with a 60-ml syringe and subsequently filtered through pre-combusted (450°C for 5 h) 25 mm GFF filters (0.7 μm pore size). For DOC measurements, 40 ml of sample were filtered and transferred to pre-cleaned 40-ml vials (Eagle-Picher®, Level 1). Samples were acidified (pH <2) with Ultrex HCl (J.T. Baker, Baker Instra-analyzed®) and refrigerated prior to analysis. All DOC measurements were made within 14 days of sampling. Previous work in our lab has shown that samples held under these conditions are stable for at least 60 d. Ultrapure water from a Milli-Q water system (Millipore Corp., Bedford, MA, USA) was used to prepare all blanks and potassium biphthalate check standards. Concentrations were calculated using a 5 point calibration curve that was verified using a secondary standard. The calibration curve was verified by generating a second 5 point curve using a secondary potassium biphthalate standard (different manufacturer), and comparing the slopes of the two curves. The slope of the second curve was within 5% of the primary curve, verifying that our initial calibration curve was correct. This method has a detection limit of 0.1 mg l^-1^, after every 15 samples an internal check standard and a blank were analyzed. Check standard values were within 5% of nominal and blanks indicated no sample carry-over. All glassware used for filtration was combusted (450°C for 5 h) prior to use in an experiment.

Maximum rate of photosynthesis (P_max_) and dark respiration (R_d_) of *Z. marina* was quantified using oxygen evolution measurements made with a Hansatech OxyLab® oxygen electrode photosynthesis system (Hansatech Instruments Ltd., Norfolk, UK.). This is a Clarke-type electrode enclosed in a temperature-controlled water jacketed reaction chamber with irradiance provided by a red light emitting diode (LED) source. Seagrass plants in chambers were subjected independently to light, temperature and salinity treatments for 72 h prior to photosynthetic measurements i.e., photosynthetic measurements took place after plants were removed from chambers at the end of the hydroponic experiment. Leaf segments from 2–3 replicate plants (about 2 cm^2^) were excised from the middle of the second leaf. Change in oxygen concentration was measured at two light levels (0 and 600 μmol photons m^-2^ s^-1^) to estimate R_d_ and P_max_. Incubations were conducted for less than 60 min and no carbon source was added to the chamber. Previous work indicates that *Z. marina* is generally light saturated at irradiance above 100 μmol photons m^-2^ s^-1^[12]. P_max_ and R_d_ were converted from oxygen normalized units to carbon units assuming a photosynthetic quotient of 1 [5,40]. Replicates were subsequently averaged and SD determined.

For comparison of photosynthetic parameters between this study and previous work, I utilized previously summarized data [12]. Previous researchers have used a variety of units to report photosynthetic parameters (P_max_ and R_d_) derived from O_2_ evolution methods. In order to compare the estimated fraction of gross primary production lost as DOC between this study and others, literature photosynthetic estimates were converted to carbon units. I used empirically derived conversion factors (6.7 g fresh g dry ^-1^, 4.4 dm^2^ g dry^-1^ and 10.6 mg chlorophyll g dry^-1^) from the local Yaquina Bay population [18,28] to convert literature derived photosynthetic units to μmol O_2_ gdw^-1^ h^-1^. Few studies provide unit conversion factors for measurements which necessitated use of local empirical values. Literature derived gross photosynthetic rates were converted to carbon units assuming a photosynthetic and respiratory quotient of 1 (1 mol O_2_ = 1 mol C). DOC exudation rates for both leaf and RR tissues were expressed as a percentage of hourly GPP (see below). Additionally, DOC exudation rates from previous studies were expressed with comparable units of mg C gdw^-1^ h^-1^ using data on chamber size and plant biomass from the original publication. Conversions explicitly assume that all DOC is derived from the seagrass.

### Experimental designs

Experiment 1 (January 2007) examined how *Z. marina* DOC exudation was effected by light (Table 1). The zero light treatment was maintained by enclosing the treatment cabinet in opaque black plastic sheeting. Intermediate and high light levels were maintained using either a 400 W (~100 μmol photons m^-2^ s^-1^, equivalent to ~ 4.3 mol photons m^-2^ d^-1^) or 1000 W (~400 μmol photons m^-2^ s^-1^, equivalent to ~ 19 mol photons m^-2^ d^-1^) metal halide lamp (Sunlight Supply, Inc., Vancouver, WA.), respectively, suspended over the chambers and maintained on a 12:12 light: dark cycle using timers. Down-welling irradiance was determined at the bottom of the tank using a LI-4000 and a 2π sensor (LI-COR, Lincoln, Nebraska). For each of the 3 light treatments a single control was incubated and sampled for DOC concentration at the same time as treatment chambers, for analysis data from all 3 controls were pooled. Seawater flowing around the chambers was used to maintain temperature. P_max_ and R_d_ measurements were made at a temperature of 12°C and salinity of 35.

**Table 1:** Summary of experimental culture conditions and biomass (mean ± SD) used during this study

**Experiment**	**Treatment**	**Light level (μmol m**^**-2**^ **s**^**-1**^**)**	**Salinity**	**Temp. (°C)**	**NH**_**4**_^**+**^**(μM)**	**Leaf Biomass (gdw chmbr**^**-1**^**)**	**RR Biomass (gdw chmbr**^**-1**^**)**
Expt. I Light	Zero	0.005	20	9	20	0.7 ± 0.2	0.3 ± 0.1
	400 W	100	20	9	20	1.0 ± 0.3	0.3 ± 0.1
	1000 W	430	20	9	20	0.9 ± 0.3	0.2 ± 0.1
Expt. II Temp	Cold	400	20	2	20	1.4 ± 0.7	0.5 ± 0.2
	Ambient	400	20	8	20	1.3 ± 0.4	0.4 ± 0.1
	Warm	400	20	15	20	1.6 ± 0.2	0.5 ± 0.2
Expt. III Salinity	Low	400	10	9	20	1.0 ± 0.3	0.3 ± 0.1
	Medium	400	20	9	20	0.9 ± 0.2	0.3 ± 0.1
	High	400	30	9	20	1.2 ± 0.3	0.4 ± 0.1

The second experiment to evaluate the effect of temperature on *Z. marina* DOC exudation was conducted during February 2007 experimental conditions are described in Table 1. Cold treatment (2°C) was maintained by insulating the water jacket surrounding the acrylic chambers and cooling it with ice. Ambient temperature (8°C) was maintained using flow-through seawater and a warm temperature treatment (15°C) was maintained by keeping the water jacket at room temperature. Temperatures in chambers were monitored using a stem thermometer and were maintained within ± 2°C of the target value. Sampling and analysis of controls as well as maintenance of light and salinity conditions were as described above. P_max_ and R_d_ were measured at a temperature of 9.5°C and a salinity of 35.

The effect of salinity on DOC exudation was evaluated during April 2007, experimental conditions are presented in Table 1. AFSW medium at salinity levels of 10, 20 and 30 were created using Red Sea Salts® dissolved in reverse osmosis (RO) water. RO water was used because of a malfunction with the Milli-Q water system. Sampling and analysis of controls as well as maintenance of light and salinity conditions were as described above. P_max_ and R_d_ were measured at temperature of 12°C and salinity = 35.

### Calculations and statistical analysis

The rate of DOC exudation was calculated using concentration data corrected to chamber volume for each compartment, then normalized to biomass and regressed against time for each treatment. Calculations were carried out independently for above-ground (leaf) tissue and below-ground rhizome and root (RR) tissue. Below-ground DOC exudation was normalized to RR tissue weight. The slope of the resulting line has rate units (i.e., mg C gdw^-1^ h^-1^) and the null hypothesis was that the slope of the line was not significantly different from zero based on an F-test with α = 0.05.

For each experiment I used one way ANOVA to evaluate treatment effects on P_max_ and R_d_ parameters. When there were no significant differences between experimental treatments, data were pooled and pooled means ± SD were used in calculating exudation as a percentage of gross primary production. Carbon normalized gross primary production (GPP) rate was calculated using equation 2.

(2) $$GPP=P_{\max}+\left|R_{d}\right|$$

To express DOC exudation as a percentage of GPP, DOC exudation was divided by GPP and multiplied by 100.

For each individual plant (n = 4 per treatment), I calculated exudation rates for leaf and RR tissue separately over the first 24 h (described above). After evaluating data for assumptions of normality and homogeneity of variance, I used ANOVA to assess differences between treatments [41,42]. In several cases, data did not conform to parametric assumptions and non parametric Kruskal-Wallis ANOVA on Ranks was used. Power analysis, indicates that experimental replication is sufficient to detect differences between treatments > 1 mg C gdw^-1^ h^-1^, which was the expected magnitude of response. When there were no significant differences between treatments, data were pooled by tissue type and compared (leaf vs RR) using ANOVA. The same analysis methods were used to assess the ratio of DOC exudation to mean primary production. All analyses were carried out using SigmaPlot 12.0 (Systat Software Inc., San Jose, CA, USA) and differences were assessed with α = 0.05.

## Results

### Effect of light intensity

**Figure 2:**
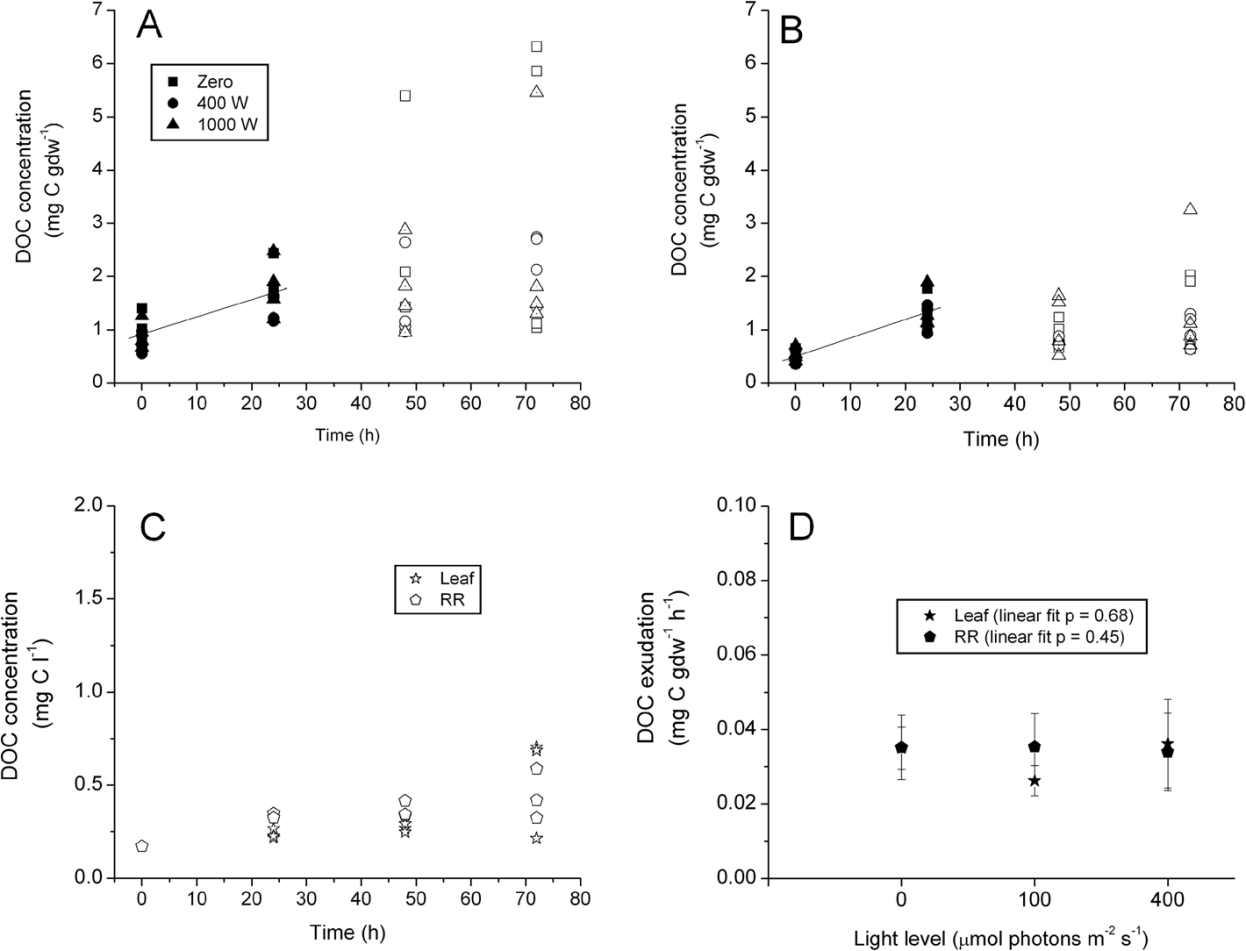
**Light Experiment.** Plot and regression analysis of DOC concentration (mg C gdw^-1^) versus time for leaf (**A**) and rhizome + root (**B**) tissues pooled over treatments. Treatments are denoted by symbol shape; open symbols were not included in the regression. DOC concentration (mg C l^-1^) versus time for the control chambers (**C**) pooled over light treatments. Leaf and Rhizome + Root DOC exudation rate (**D**) versus irradiance; linear regressions were fit to the data, probability values are presented. Note difference in units and Y-axis scaling between plots.

There were no significant differences among DOC exudation rates for experimental light treatments either for leaf (ANOVA df = 2, F = 1.84, P = 0.213) or RR (ANOVA df = 2, F = 0.02, P = 0.975) tissue. Pooled leaf and RR DOC exudation rates were virtually identical at 0.032 and 0.034 mg C gdw^-1^ h^-1^(ANOVA df = 1, F = 0.473, P = 0.499); regression analysis indicates that these were significant relationships with R^2^ values of 0.58 and 0.80, respectively (Figure 2A & B, Table 2). DOC concentration in the upper portion of control chambers ranged between 0.2 and about 0.25 mg C l^-1^, while those in below-ground controls were between 0.2 and 0.3 mg C l^-1^ during the first 24 hours (Figure 2C). DOC increase in control chambers was an order of magnitude lower than treatment rates at 0.003 mg C l^-1^ h^-1^ over the first 24 h; however, variability between replicates increased with time (Figure 2C). Regression analysis indicated that there was no significant linear relationship between *Z. marina* DOC exudation and light treatment (Figure 2D).

**Table 2:** **Summary of pooled DOC exudation rates (mg C gdw**^**-1**^ **h**^**-1**^**) calculated by linear regression from experimental chambers**

**Expt.**	**Treatment**	**Tissue**	**Regression coefficients**					**ANOVA**
			**Rate**	**SD**	**R**^**2**^	**n**	**P**	
Light	Pooled	Leaf	0.032	0.024	0.583	24	<0.001	a
	Pooled	RR	0.034	0.019	0.805	24	<0.001	a
Temp.	Pooled	Leaf	0.034	0.029	0.576	24	<0.000	a
	Pooled	RR	0.024	0.014	0.754	24	<0.000	b
Salinity	Pooled	Leaf	0.069	0.088	0.396	24	0.000	a
	Pooled	RR	0.045	0.063	0.345	24	0.002	a

Estimated P_max_ and R_d_ were variable within and among treatments but ANOVA indicated that there were no significant differences between light treatments for either parameter (P > 0.05). Pooled mean P_max_ and R_d_ (Table 3) were used to calculate exudation as a percentage of GPP. Mean leaf DOC exudation rates accounted for about 1.4% of GPP, while RR exudation accounted for *ca.* 1.5% of GPP (Table 3). ANOVA indicated that the ratio of DOC to primary production (DOC:PP) also did not show any significant treatment effects (df = 2, F =1.84, P = 0.213 and df = 2, F = 0.02, P = 0.975 for leaf and RR tissue respectively); likewise there was no significant difference between tissues (df = 1, F = 0.473, P = 0.499).

**Table 3:** **Summary of mean net P**_**max**_**and R**_**d**_**(μmol C gdw**^**-1**^ **h**^**-1**^**) ± SD and estimated DOC exudation rates expressed as a percentage of GPP**

**Experiment**	**P**_**max**_	**R**_**d**_	**n**	**Leaf exud**	**RR exud**
	**(μmol C gdw**^**-1**^ **h**^**-1**^**)**	**(μmol C gdw**^**-1**^ **h**^**-1**^**)**		**(% GPP)**	**(% GPP)**
Light	165 ± 94	−28 ± 33	9	1.4 ± 0.7	1.5 ± 0.6
Temp	106 ± 36	−14 ± 8	6	2.4 ± 1.8	1.7 ± 0.9
Salinity	236 ± 138	−20 ± 12	5	2.2 ± 1.2	1.5 ± 0.9

### Effect of temperature

**Figure 3:**
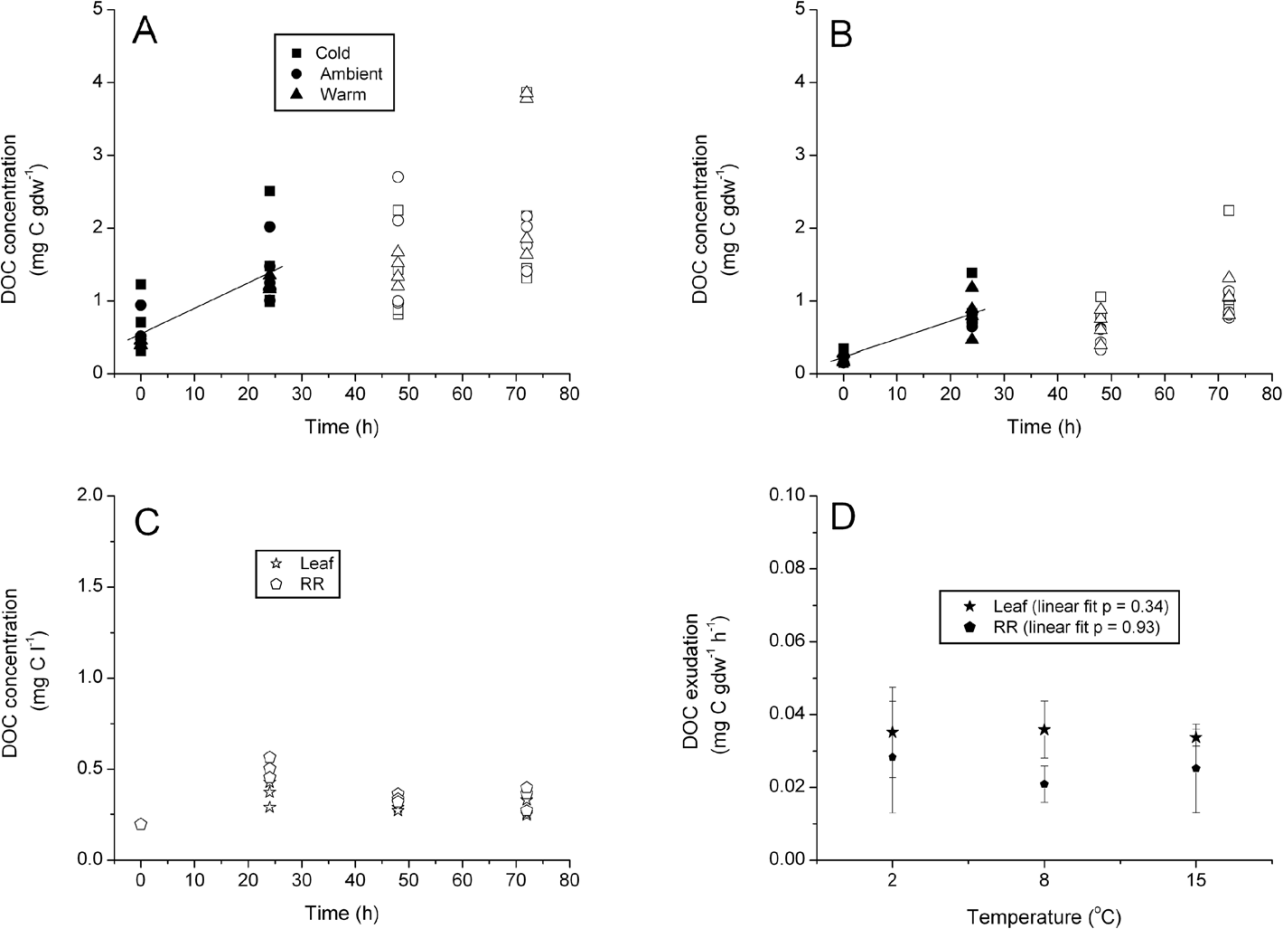
**Temperature Experiment.** Regression analysis of DOC concentration (mg C gdw^-1^) versus time for pooled leaf (**A**) and rhizome + root (**B**) tissues, pooled over treatments. Treatments are denoted by symbol shape; open symbols were not included in the regression. DOC concentration (mg C l^-1^) versus time for the control chambers (**C**) pooled over treatments. Leaf and Rhizome + Root DOC exudation rate (**D**) versus temperature; linear regressions were fit to the data, probability values are presented. Note difference in units and Y-axis scaling between plots.

There were no significant differences among DOC exudation rates for experimental temperature treatments either for leaf (ANOVA df = 2, F = 0.066, P = 0.936) or RR (df = 2, F = 0.397, P = 0.684) components. Pooled leaf DOC exudation rate of 0.034 mg C gdw^-1^ h^-1^ was about 30% higher (ANOVA on ranks df = 1, H = 7.68, P = 0.006) than pooled RR exudation rate of 0.024 mg C gdw^-1^ h^-1^ (Figure 3A & B, Table 2). DOC concentrations in control chambers ranged between about 0.2 and about 0.5 mg C l^-1^ in both the leaf and RR compartments during the first 24 h (Figure 3C). DOC increase in control chambers was an order of magnitude lower than treatment rates at 0.006 mg C l^-1^ h^-1^ over the first 24 h. Control DOC concentrations decreased between the 24 h and 48 h sampling periods (Figure 3C). Regression analysis indicated that there was no significant linear relationship between *Z. marina* DOC exudation rates and temperature (Figure 3D).

Treatment P_max_ and R_d_ values were variable; ANOVA indicated that differences between treatments were not significant (P > 0.05). Data were pooled and mean ± SD (Table 3) was used to estimate exudation as a percentage of GPP. Mean leaf DOC exudation rates accounted for about 2.4% of GPP, while RR exudation accounted for another 1.7% (Table 3). ANOVA indicated that the DOC:PP also did not show any significant treatment effects (df = 2, F =0.06, P = 0.936 and df = 2, F = 0.397, P = 0.684 for leaf and RR tissue respectively); however, ANOVA on ranks indicated there was a significant difference between tissues (df = 1, H = 7.680, P = 0.006), with leaf tissue having greater DOC:PP than RR tissue (Table 2).

### Effect of salinity

**Figure 4:**
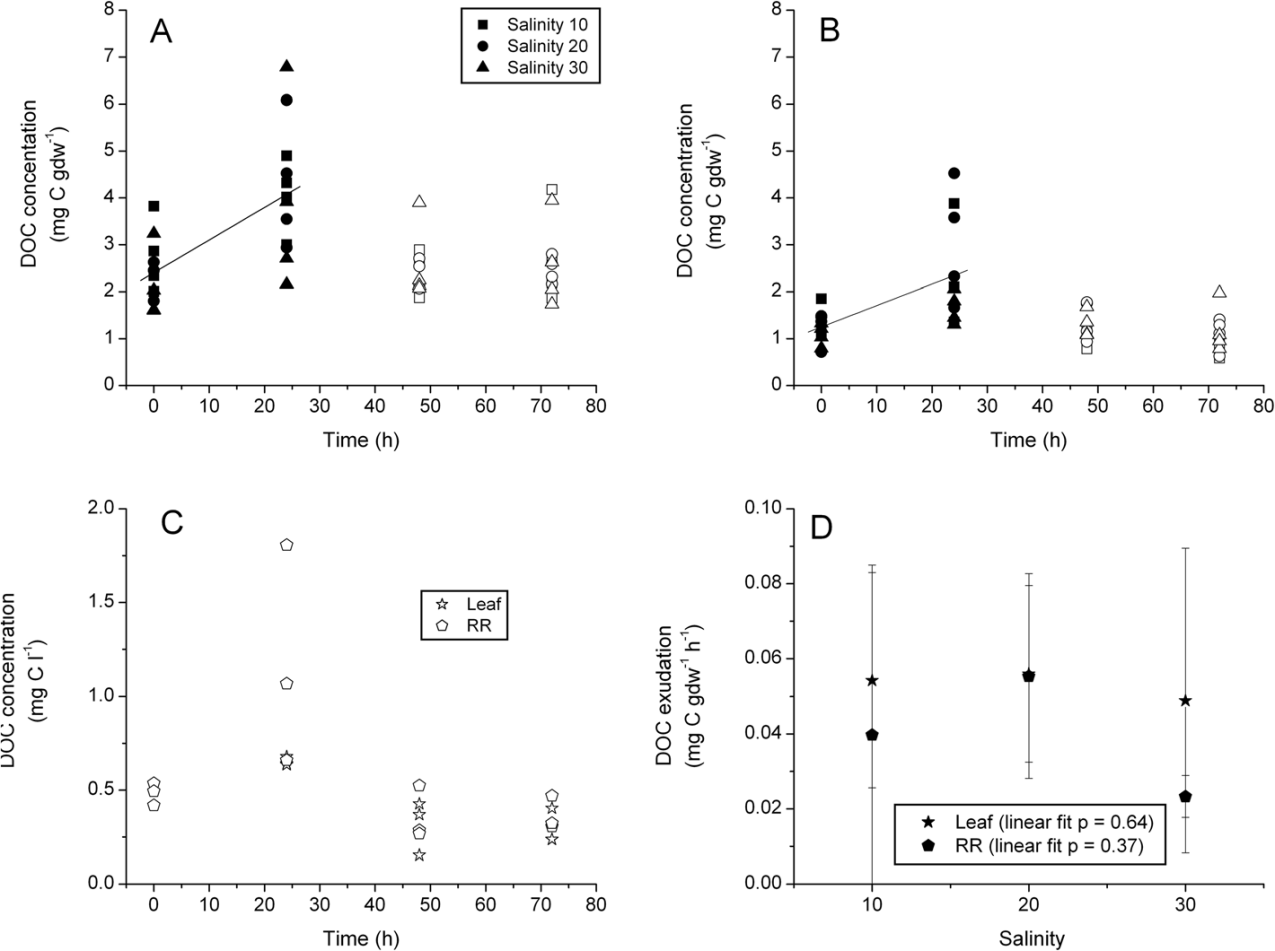
**Salinity Experiment.** Regression analysis of DOC concentration (mg C gdw^-1^) versus time for pooled leaf (**A**) and rhizome + root (**B**) tissues, pooled over treatments. Treatments are denoted by symbol shape; open symbols were not included in the regression. DOC concentration (mg C l^-1^) versus time for the control chambers (**C**) pooled over treatments. Leaf and Rhizome + Root DOC exudation rate (**D**) versus salinity; linear regressions were fit to the data, probability values are presented. Note difference in units and Y-axis scaling between plots.

There were no significant differences among DOC exudation rates for experimental salinity treatments either for leaf (ANOVA df = 2, F = 0.32, P = 0.733) or RR (df = 2, F = 2.02, P = 0.188) components. Pooled leaf DOC exudation rate was 0.069 mg C gdw^-1^ h^-1^, and was not significantly different (ANOVA on ranks df = 1, H = 2.25, P = 0.133) from exudation rate of pooled RR (Figure 4A & B, Table 2). DOC concentrations in control chambers ranged between 0.5 and about 1.8 mg C l^-1^ during the first 24 h and subsequently decreased to less than 0.5 mg C l^-1^ by the 48 h sampling (Figure 4C). DOC increase in control chambers was an order of magnitude lower than treatment rates at 0.005 mg C l^-1^ h^-1^ over the first 24 h. Linear regression indicated that there was no significant relationship between DOC exudation rates and salinity (Figure 4D). Measured P_max_ and R_d_ showed considerable variability among salinity treatments but differences were not statistically significant (P > 0.05) and treatment values were pooled (Table 3). Mean leaf DOC exudation accounted for 2.2% of GPP, while RR exudation accounted for 1.5% of GPP (Table 3). ANOVA indicated that the DOC:PP also did not show any significant treatment effects (df = 2, F =0.322, P = 0.733 and df = 2, F = 0.810, P = 0.475 for leaf and RR tissue respectively); likewise ANOVA on ranks indicated there was no significant difference between tissues (df = 1, H = 0.083, P = 0.773).

## Discussion

Contrary to expectations, measured DOC exudation rates were not strongly correlated with light, temperature or salinity treatments. Measured *Z. marina* DOC exudation, expressed as a rate or as a percentage of GPP, was consistent with previous measures despite differences in methods, locations and species. Field studies have reported seagrass DOC exudation rates ranging between 0.007 and 0.125 mg C gdw^-1^ h^-1^, with most values on the order of 0.019 to 0.057 mg C gdw^-1^ h^-1^ (Table 4). Measured hydroponic DOC exudation rates were comparable with leaf exudation rates between 0.032 and 0.069 mg C gdw^-1^ h^-1^ and rhizodeposition rates between 0.024 and 0.045 mg C gdw^-1^ h^-1^ (Table 4). Measured photosynthetic characteristics were also comparable to other studies, but P_max_ was near the low end of the range while R_d_ was near the middle of the range of reported values (Table 5, converted values). Similarity of measured physiological rates indicates that hydroponic chambers can be useful experimental model systems for short term (*e.g.*, hours to days) physiological experiments and that *Z. marina* DOC exudation rates may be insensitive to modest fluctuations of environmental drivers over periods of less than 24 h. These findings are consistent with previous work suggesting that there is little coupling between *Z. marina* production and the sediment microbial community [16]. DOC exudation rates may be related to species specific attributes, since several tropical species can influence environmental DOC concentrations [17-19,24-26]. 

**Table 4:** Comparison of literature values for DOC exudation by seagrasses expressed as rates and as a percentage of gross primary production (GPP)

**Seagrass**	**Exudation rates (mg C gdw**^**-1**^ **h**^**-1**^**)**	**Leaves (%)**	**Rhizome/Root (%)**	**Source**
*Thalassia testudinum*	0.035 – 0.125			[26]^a^
		1	15-30	[55]
			~1	[14]
	0.019 ± 0.003^b^	1.3		[13]
*T. hemprichii*			5.4	[20]
*Halodule wrightii*		1	6-17	[21]
	0.022 ± 0.008^b^			[13]
*Posidonia oceanica*	0.022	1.9		[25]
	0.007 - 0.02			[19]^c^
*Cymodocea rotundata*			7	[20]
*Cymodocea nodosa*	0.057			[17]
*Zostera marina*		2		[15]
		0.2	2.2	[14]
	0.015 ± 0.006^b^			[59]
	0.032 -0.069	0.6 -4.2		leaf
	0.024 – 0.045		0.6 – 2.4	rhizome

^a^ DOC flux from [26], above ground biomass ~100 gdw m^-2^ from [49].

^b^ mean ± SD.

^c^ values based on conversion of annualized (4360 mmols C m^-2^ y^-1^) and June (34.7 mmols C m^-2^ d^-1^) fluxes.

**Table 5:** ***Zostera marina *photosynthetic parameters summarized and converted to “normalized units” using empirically derived conversion factors**

**Original units**	**Converted P**_**max**_	**Converted R**_**d**_	**Original**
	**μmol C gdw**^**-1**^ **h**^**-1**^	**μmol C gdw**^**-1**^ **h**^**-1**^	Reference*
μmol O_2_ gfw^-1^ min^-1^	200 -680	nd	[45]
μmol O_2_ dm^-2^ min^-1^	174	nd	[53]
μmol O_2_ gdw^-1^ min^-1^	72 - 90	51 - 82	[54]
μmol O_2_ dm^-2^ min^-1^	528	nd	[55]
mg O_2_ gdw^-1^ h^-1^	313 - 388	nd	[56]
μmol O_2_ mg chl^-1^ min^-1^	253	3.7 - 19	[47]
μmol O_2_ mg chl^-1^ min^-1^	317	51	[57]
μmol O_2_ gdw^-1^ h^-1^	nd	27-51	[58]
Range	174 - 528	3.7 - 82	

*As cited in [12].

Abbreviations: *gfw* gram fresh weight, *gdw* gram dry weight, *mg chl* milligram chlorophyll, *dm* decimeter, *min* minute, *h* hour.

It is well known and accepted that light, temperature and salinity influence seagrass physiological rates such as C fixation, respiration and cellular osmotic pressure. Lack of rapid response in DOC exudation rates, from either leaf or RR tissue suggests that this loss term may be a passive process constrained by physical or diffusion barriers such as the cuticle or suberized, lignified below-ground tissues [43]. Terrestrial plants use a variety of mechanisms to control loss of water vapor aquatic plants may use similar structures to minimize leakage of organic compounds to the environment.

DOC concentrations from control chambers were low relative to experimental treatments; indicating that there was no serious DOC contamination issues during any of the experiments. In general, there was a slight increase in the DOC concentration of controls during the first 24 h sampling interval; however, when expressed as a rate, these increases were an order of magnitude smaller than rates from treatment chambers. Increased DOC in the controls may have been related to splashing from treatment chambers, dust, aerosol deposition or a result of hydrocarbons in the air supply. Irrespective of the source, these accumulation rates were not sufficient to account for measured rates of DOC increase in treatment chambers. Additionally, in two experiments there was a rapid decrease in the DOC concentration between 24 h and 48 h sampling periods, while in the other experiment variability between controls increased. Together, this suggests that a loss mechanism, such as bacterial mineralization had become influential. Marine bacteria doubling times are dynamic [15,44], but a reasonable rule of thumb is that they double about 1 d^-1^[39] which could explain this pattern. Based on the control data, bacterial consumption of DOC is a reasonable explanation for the observed decrease in DOC after 24 h of incubation in these experiments and provides justification for calculating exudation rates from the first two sampling points. As a result, DOC exudation rates presented here should be considered first-order estimates.

### DOC exudation in relation to environmental drivers

Light, temperature and salinity treatments applied in these experiments were similar to the range of conditions these plants encounter in outer coast estuaries of the Pacific Northwest, USA. Similarity in measured DOC loss rates within and between experiments (Table 2), may be a result of the plants being adapted to fluctuating local environmental conditions (*e.g.*, adaptation or acclimation) or of physical processes (*e.g.*, diffusion) controlling passive DOC exudation. Additional experiments will be required to determine if *Z. marina* DOC exudation responds to extreme stress (light, temperature or salinity) outside the “normal” range of variation encountered.

Light availability is directly correlated with carbon fixation via photosynthesis. Consequently, I expected that DOC exudation in the light treatments would be greater than in the dark, especially since carbon transport between leaf and rhizome tissue has been shown to be light dependent [45]. Light treatments used in this experiment were at or above the saturating irradiance (*ca.* 100 μmol photons m^-2^ s^-1^) but well below levels leading to photoinhibition [12] and since photosynthesis requires light energy these plants fixed more carbon than plants in the dark treatment. The zero light treatment is a regular occurrence for some *Z. marina* populations, which may experience extended periods (*e.g.*, weeks) of darkness during winter storms [6,28]. The lack of correlation between light availability and DOC exudation suggests that carbon fixation via photosynthesis may be de-coupled from exudation or that loss occurs at a fixed rate via a physical process.

Temperature has a fundamental impact on all metabolic processes through its influence on enzyme kinetics. In the temperature experiment, plants were exposed to conditions from 2 to 15°C, which is within the normal range of temperatures for *Z. marina*[28,46,47]. I expected that a 13°C temperature range would alter exudation rates if it were an active transport process or by influencing photosynthetic production. Alternatively, a passive transport mechanism (*e.g.*, diffusion) for DOC, constrained by physical permeability of the plant epidermis would be less influenced by temperature. And as previously discussed, light did not influence exudation in these experiments.

Seagrasses in general exhibit a variety of mechanisms for acclimating to salinity variations that range from changes in cellular ion concentrations to elasticity of the cell wall [48]. Rapid salinity responses generally include osmotic adjustments of inorganic ions and organic osmolytes such as proline, carbohydrates and organic acids [48]. Consequently, salinity would be expected to affect internal plant constituents which were not evaluated in this study. However, this may not impact DOC exudation rates if exudation were a passive diffusion process. Extreme salinity events (*e.g.*, exposure to freshwater or hypersalinity) with bounds outside the normal range to which this population is exposed would likely produce different results than observed.

### Leaf versus rhizodeposition

Although differences were generally not statistically significant, in two of the three experiments *Z. marina* leaf DOC exudation was at least 30% higher than rhizodeposition rates. In contrast, for tropical species with high root:shoot ratios, other studies concluded that rhizodeposition exceeded leaf exudation (Table 4). Hydroponically measured rhizodeposition rates (0.6 to 2.4% GPP) are similar to the only estimate of *Z. marina* rhizodeposition in the primary literature 2.2% GPP [14].

A number of factors may influence the relative exudation rates of the different plant tissues such as “leakiness”, biomass allocation or chamber artifacts. Anatomical differences between leaf and RR tissues likely influence DOC loss rates. Leaf epidermal cells have a thin cuticle that may be more permeable to DOC than thickened, lignified, suberized epidermal cells of RR tissue [43]. Biomass allocation may be correlated with rhizodeposition, such that large amounts of biomass, slowly leaking DOC result in a build-up of exudates. Since, below-ground tissues store non-structural carbohydrates, the relative proportion of above to below-ground biomass (root:shoot ratios) may influence the amount of DOC lost to sediments. For example, 80-90% of *T. testudinum* biomass (total biomass = 700–1500 gdw m^-2^) occurs in the below-ground fraction [49], while in *Z. marina* from Yaquina Bay only 20-40% of biomass (total biomass = 50–200 gdw m^-2^) is below-ground [28, Kaldy, unpublished data]. Since rhizodeposition is normalized to biomass it is not surprising that *Z. marina* would have lower below-ground exudation potential. Alternatively, the oxic hydroponic environment that below-ground tissues experienced within the chambers was very different from the highly reduced, anoxic sediments these tissues typically inhabit. This likely reduced the amount of time that below-ground tissues utilize the glycolosis pathway for energy production and may have reduced the build-up of organic by-products [50]. Previous work suggests that the oxygen status of the root environment can influence DOC exudation [14]. In general, the role *in vitro* culture conditions may play in measuring seagrass exudation rates requires continued evaluation.

### Scaling

Individual *Z. marina* plants of the same stature as used in these experiments are capable of dramatically drawing down water column nutrients (Kaldy, unpublished data) and of increasing the DOC concentration in these chambers (Figures 2, 3 and 4). Consequently, biomass of individual plants (1–2 gdw) was sufficient to provide measureable DOC exudation and nitrogen uptake rates (Kaldy, unpublished data) in hydroponic chambers. Similarly, multiple leaves of *P. oceanica* were required to obtain measureable amounts of DOC in laboratory bell jar experiments [25]. When results of previous field studies are normalized to biomass, and assuming that all DOC is from seagrass, rates of DOC exudation were consistent with the hydroponic *in vivo* rates measured here (Table 4). Taken together, this suggests that DOC exudation from seagrass leaf or RR tissue is generally low for an individual plant. However there could be a synergistic effect of many plants in a meadow producing low levels of DOC that may explain the observed diel pattern of field water column DOC concentrations [17,19,26]. Alternatively, Velimirov [25] suggests that fresh DOC directly exuded from seagrass is a minor contributor to the DOC pool and that diel patterns of DOC sometimes observed in the field may be the result of complex interactions between plants and sediment carbon pools. Consequently, scaling associated with leaf and RR biomass may be an important consideration for interpreting the contribution of individual DOC release relative to whole populations. Additionally, DOC losses may be dependent on the species being studied due to species specific attributes [16,51].

## Conclusions

Although these hydroponically derived DOC exudation rates were estimated from an experimental design that was pseudoreplicated [31] they do provide first order estimates of whole plant DOC exudation that are consistent with previous field studies. Additionally, as suggested by Oksanen [32], these experiments provide testable hypotheses for future research. Specifically, this preliminary work suggests that seagrass DOC exudation may be a passive process controlled by physical, diffusive constraints and not an active transport process that responds quickly to environmental variations. These preliminary empirical estimates of exudation and rhizodeposition can provide first-order estimates for complex mechanistic seagrass models [23,52]. Furthermore, hydroponic chamber systems provide a useful model system for short-term seagrass physiological experiments lasting hours to days. Future studies need to consider interactive effects of multiple stressors which have been largely ignored in seagrass physiological studies and stress conditions that exceed the normal experience of the test population.

## Competing interests

The author declares that he has no competing interests.

## Authors’ contributions

JEK conceptualized, planned and executed all phases of these experiments and developed the manuscript.
